# Probe-Specific Procedure to Estimate Sensitivity and Detection Limits for ^19^F Magnetic Resonance Imaging

**DOI:** 10.1371/journal.pone.0163704

**Published:** 2016-10-11

**Authors:** Alexander J. Taylor, Josef Granwehr, Clémentine Lesbats, James L. Krupa, Joseph S. Six, Galina E. Pavlovskaya, Neil R. Thomas, Dorothee P. Auer, Thomas Meersmann, Henryk M. Faas

**Affiliations:** 1 Sir Peter Mansfield Imaging Centre, School of Medicine, University of Nottingham, Nottingham, United Kingdom; 2 Institute of Energy and Climate Research, Fundamental Electrochemistry (IEK-9), Forschungszentrum Juelich GmbH, Juelich, Germany; 3 School of Chemistry, University of Nottingham, Nottingham, United Kingdom; Universitatsklinikum Wurzburg, GERMANY

## Abstract

Due to low fluorine background signal *in vivo*, ^19^F is a good marker to study the fate of exogenous molecules by magnetic resonance imaging (MRI) using equilibrium nuclear spin polarization schemes. Since ^19^F MRI applications require high sensitivity, it can be important to assess experimental feasibility during the design stage already by estimating the minimum detectable fluorine concentration. Here we propose a simple method for the calibration of MRI hardware, providing sensitivity estimates for a given scanner and coil configuration. An experimental “calibration factor” to account for variations in coil configuration and hardware set-up is specified. Once it has been determined in a calibration experiment, the sensitivity of an experiment or, alternatively, the minimum number of required spins or the minimum marker concentration can be estimated without the need for a pilot experiment. The definition of this calibration factor is derived based on standard equations for the sensitivity in magnetic resonance, yet the method is not restricted by the limited validity of these equations, since additional instrument-dependent factors are implicitly included during calibration. The method is demonstrated using MR spectroscopy and imaging experiments with different ^19^F samples, both paramagnetically and susceptibility broadened, to approximate a range of realistic environments.

## Introduction

^19^F MRI is an attractive modality for a range of applications including molecular sensing [1,2] and cell tracking [3–5]. Imaging of inflammation *in vivo* using perfluorocarbon (PFC) emulsions has been particularly promising, allowing visualisation of inflammation in the peripheral nervous system in rats [6] as well as the immune response during oncolytic virotherapy in a mouse tumor model [7].

^19^F nuclei are intrinsically sensitive due to their high gyromagnetic ratio, and the absence of a detectable fluorine background signal *in vivo* offers high specificity. However, the limited amount of fluorine that can be delivered to a target tissue, and consequently the low signal-to-noise ratio (SNR) combined with the low SNR efficacy [8]–i.e. the minimum concentration required to generate a significant SNR within a reasonable imaging time–is the major obstacle for the wider application of fluorine MRI [9].

*In vivo*, the quantification of local fluorine concentration can be achieved by comparing the total signal of a sample with reference standards of known concentration [10], since the fluorine signal is proportional to the number of ^19^F spins. However, it would be desirable to estimate the potential sensitivity of a ^19^F MRI set-up even before a pilot experiment. This could be the case for new fluorine based contrast agents, drugs or molecular ^19^F sensors during the experimental design phase, when complex synthesis would be required before any experiment could be carried out. Enzymatic sensors [11,12] for example rely on the interaction of the fluorine signal with a paramagnetic metal that induces a paramagnetic shift or relaxation. Even once synthesized, the actual fluorine based molecule is often only available in small quantities and a complete pilot experiment may not be initially possible.

In order to determine the potential sensitivity of such agents, we propose a method that allows an estimation of the minimum ^19^F concentration required to achieve a target SNR in a ^19^F experiment with a particular setup by performing a calibration using readily available fluorine compounds. The theoretical framework for determining the sensitivity in a MRI experiment is well established by Hoult and Richards [13]. It was further extended to account for imaging *in vivo* with additional factors including electrical losses [14], additional noise contributions [15,16], coil design [17] and field strength [18]. Based on these equations we introduce a calibration factor that is specific for the experimental imaging setup and allows the determination of the minimum detectable concentration. The physical relevance of this calibration factor is explained by using textbook equations, yet it is noted that various additional factors are implicitly accounted for by the experimental calibration with a known compound. An exception is the dephasing time constant of transverse magnetization, *T*_2_*, which can vary considerably in different environments. It is shown that *T*_2_* for fluorine can be obtained through extrapolation from the *T*_2_* value of ^1^H.

We will show that this framework provides a way to estimate the sensitivity of a ^19^F experiment using a cheap, readily available model compound and extrapolate this to another, possibly expensive or valuable ^19^F compound. The framework we present here is demonstrated for simple pulse-acquire magnetic resonance spectroscopy (MRS) and fast low angle shot (FLASH) imaging, but can also be the basis for extrapolation to other pulse sequences.

## Materials and Methods

### Experimental factors contributing to sensitivity

Generally, the sensitivity in magnetic resonance (MR) can be divided into separate aspects: the sample, the instrument hardware, the pulse sequence and the data analysis protocol. Although recent developments in MR methodology have somewhat blurred the lines between these four aspects, they can be treated, to a good approximation as multiplicative and can therefore be discussed independently of each other.

The sample behaviour can be predicted very well, within the high temperature limit, with the number of spins contributing to the signal as central variable accessible to the experimenter. The choices available for the pulse sequence and analysis protocol are manifold and their influence on the sensitivity can be substantial. This influence can be quantified well using analytical and numerical simulations tools. Finally, the hardware represents a parameter with a large influence on the sensitivity, which is generally only accessible via calibration measurements and cannot be easily modified or changed in most medical applications. To estimate the concentration of a sample, a one-time hardware calibration is suggested, while simple pulse sequences and straightforward analysis routines are considered. Effects of more advanced excitation and analysis techniques could be included by taking advantage of the multiplicity of their influence, but respective procedures are only outlined.

### Theoretical SNR

We define the signal-to-noise ratio as the ratio of the peak amplitude of the nuclear magnetic resonance (NMR) signal divided by the root mean square (rms) noise amplitude. As detailed in S1 Text, the theoretically achievable SNR, *Ψ*, in a single pulse nuclear magnetic resonance experiment can be approximated by

$$\Psi =\frac{cN_{A}n_{e}\gamma \hbar^{2}V_{s}}{16}\sqrt{\frac{K^{2}{µ}_{0}Q\omega_{0}^{3}\pi T_{2}^{*}}{V_{c}Fk_{B}^{3}T^{3}}}\sin\left(\beta \right).$$ [1]

Here, in SI units, *c* is the concentration in units of mol/m^3^, *N*_*A*_ the Avogadro constant, *n*_*e*_ the number of equivalent fluorine spins per molecule with the same chemical shift, γ the gyromagnetic ratio, ħ the reduced Planck constant, *V*_*s*_ the sample volume, *k*_*B*_ the Boltzmann constant, *T* the temperature, *T*_2_* the dephasing time constant of transverse magnetization, *V*_*c*_ the radiofrequency (rf) coil volume, *F* the pre-amplifier noise figure (which is generally constant for the frequency range covered by a given nucleus [19]), μ_0_ the permeability of free space, *Q* the quality factor of the detection circuit, *ω*_0_ the Larmor frequency of the nuclear spins, and *β* the flip angle associated with the excitation pulse. *K* is a numerical factor introduced by Hoult and Richards to account for the specific receiving coil geometry [13].

To translate this approach into practice so that a minimum concentration can be easily evaluated, we introduce a calibration factor, *Λ*, which is determined experimentally. Based on our idealized SNR equation, the calibration factor is defined as

$$\Lambda :=\sqrt{\frac{K^{2}Q}{FV_{c}}}.$$ [2]

The calibration factor has to be determined once for a given hardware and coil configuration in order to correlate the predicted and experimental SNRs. In practice, *Λ* also includes specific corrections for set-ups where SNRs differ from the idealized situation given by Eq 1. These correction factors include the coil geometry dependency of the filling factor [13] and only contribute a constant multiplicative factor for a given MRI hardware set-up. To simplify further, all constant terms are combined into a parameter, *Π*, which is independent of the sample and can be evaluated without performing an experiment:

$$\Pi :=\frac{16}{N_{A}\gamma \hbar^{2}}\sqrt{\frac{k_{B}^{3}T^{3}}{\mu_{0}\pi \omega_{0}^{3}}}.$$ [3]

An explicit equation for the threshold fluorine marker concentration required to achieve a target SNR is obtained after rearranging Eq 1:

$$c=\frac{\Pi }{\Lambda }\frac{\Psi }{n_{e}V_{s}\sqrt{T_{2}^{*}}}\frac{1}{\sin\left(\beta \right)}.$$ [4]

To account for the resulting SNR following a number N_R_ of identical pulse-acquisitions, Eq 4 must be modified to include an increased SNR, linearly dependent on $\sqrt{N_{R}}$. The longitudinal relaxation time, *T*_1_, and the repetition time, *T*_*R*_, must also be included now to account for the recovery of the magnetization after each pulse. Combining these three parameters with Eq 4 gives:

$$c=\frac{\Pi }{\Lambda }\frac{\Psi }{n_{e}V_{s}\sqrt{N_{R}T_{2}^{*}}}SF\frac{1}{\sin\left(\beta \right)},$$ [5]

where *SF* is defined as the saturation factor,

$$SF=\frac{1-\cos(\beta )exp(-T_{R}/T_{1}))}{\left(1-exp(-T_{R}/T_{1})\right)},$$ [6]

introduced to account for incomplete relaxation (*SF* ≈ 1, if *T*_*R*_ ≳ 5 × *T*_1_).

To estimate the rms noise, data points from off-resonant regions of a spectrum or an image are selected. In principle, a more reliable method would be to repeat an experiment multiple times and determine the rms noise from the variation of the signal amplitude. However, in addition to thermal noise, such a procedure also includes multiplicative noise, which does not affect the fundamental detection limit as it scales proportionally with the signal and, at low SNR values, eventually gets overtaken by thermal noise [20]. Hence the selected method provides not only a simpler, but also a more robust way to estimate detection limits by masking contributions that become dominant at high SNR values only.

The primary focus on the detection limit and the, potentially simplified, assumption of white thermal noise also minimizes the necessity to consider more advanced statistical noise characterization and filtering techniques [21]. The benefit of these methods are scanner and protocol dependent and may be included by specifying an additional factor for a particular experimental setting, yet the necessary effort may only be justified if a large number of studies with similar protocols are conducted using a particular instrument. Similarly, ^19^F ghosting artefacts are not considered using the presented method, but could be incorporated using previously published methods [22]. These artefacts may considerably affect the reliability of MR data, yet the effect on the detection limit is small.

### Noise and signal loss in magnitude transformed MR data

Many MRI and some MRS experiments use magnitude transformed images and spectra. In our analysis, we estimate the rms noise from off-resonant regions of magnitude spectra or images and use a scaling factor of 0.655 to account for the resulting Rician noise distribution where *Ψ* ≳ 3. For lower *Ψ*, the scaling factor would need to be readjusted based on the procedure outlined by Gudbjartsson et al. [23].

The dominant source of noise is assumed to be thermal, or Johnson noise. *In vivo* however physiological noise is significant, as well as the thermal noise generated by the patient or in the detection circuit [15,16]. This cannot, in general, be specified using a constant correction term, but experimental quantification is usually straightforward. Hence additional noise can be incorporated by scaling the noise term according to the added contribution with respect to the thermal noise level.

### Detection limits for MRI

To accommodate imaging experiments, *E*q 5 can be adapted to account for a sample’s signal being spread over the voxels of volume *V*_*v*_. We must also include a numerical factor specific to the sequence of interest, which represents the theoretical signal available during acquisition. This factor could be greater than one, for example in the case of a multi-spin-echo sequence.

In this example we have chosen one of the common imaging sequences used in fluorine experiments [3], a FLASH gradient echo sequence [24], whose associated numerical signal factor is (sin(*β*)exp(−*TE*/*T*_2_)), which combined with Eq 5 and the other relevant imaging factors gives:

$$c=\frac{1}{0.655}\frac{\Pi }{\Lambda }\frac{\Psi }{n_{e}V_{v}\sqrt{N_{R}\;T_{2}^{*}}}SF\frac{1}{\sin\left(\beta \right)e^{-\frac{TE}{T_{2}}}},$$ [7]

where *TE* is the echo time and *T*_2_ is the transverse relaxation time. The equation can be adapted to any sequence, by suitable replacement of the sequence specific factor. Using Eq 7 we can also express *N*_*v*_, the minimum number of detectable spins in a voxel required for a sample to achieve a target SNR:

$$N_{v}=\frac{1}{0.655}\frac{\Pi N_{A}}{\Lambda }\frac{\Psi }{\sqrt{N_{R}\;T_{2}^{*}}}SF\frac{1}{\sin\left(\beta \right)e^{-\frac{TE}{T_{2}}}}.$$ [8]

### Sample preparation

**Fig 1:**
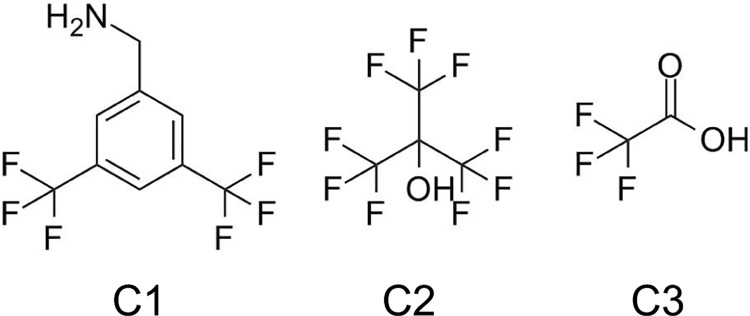
Chemical structures of compounds with variable number of fluorine spins per molecule. Compound C1 (3,5-bis(trifluoromethyl)benzylamine), six fluorine spins; C2 (perfluoro-tert-butyl alcohol), nine fluorine spins; C3 (Trifluoroacetic acid), three fluorine spins.

Two solutions were initially prepared: the first contained 58 mM of 3,5bis(trifluoromethyl)-benzylamine (C1 in Fig 1, Sigma Aldrich, UK) in methanol (Sigma Aldrich, UK); the second contained 320 mM of perfluoro-tert-butyl alcohol (Sigma Aldrich, UK) in methanol (C2 in Fig 1, Sigma Aldrich, UK). All other solutions were made by dissolving anhydrous powder of trifluoroacetic acid (TFA, C3 in Fig 1) (Sigma Aldrich, UK) in distilled H_2_O. All solutions containing C3 were made from 1 M stock solutions (compound concentration) and diluted in H_2_O.

A line broadening effect on a compound’s linewidth is likely to occur *in vivo*, where paramagnetic species or changing magnetic susceptibility affect fluorine molecules’ linewidth. In our experiments we sought to replicate such possible line broadening to verify our method’s robustness in such cases. To induce line broadening by paramagnetic relaxation, a gadolinium based contrast agent, gadobenate dimeglumine (MultiHance, Bracco Imaging, SpA, Milan, Italy) was added to C3. To induce susceptibility broadening, a C3 solution was placed in a porous medium that consisted of 0.5 mm diameter solid glass beads (BioSpec Products, Bartlesville, OK). The solution volume fraction in glass beads was derived from the packing volume of the glass beads in the NMR tube, obtained experimentally by comparing liquid volumes between an empty tube and a tube containing the glass beads (relative volume of glass beads: 60 ± 10%, compared with an ideal packing volume of 74%) [25].

### ^19^F and ^1^H NMR and MRI experiments

All experiments were performed on a 9.4 T vertical bore Bruker Avance III microimaging system (Bruker Corporation, Billerica, USA) with a 25 mm volume coil tuned to the fluorine resonance frequency of 376.5 MHz. Thin walled 5 mm NMR tubes containing 1 mL of each sample were used in all measurements. Single pulse experiments were used to measure ^19^F spectroscopic SNRs, where the pulse power and duration were calibrated manually. ^19^F images were acquired with a fast low angle shot (FLASH) sequence (echo time *TE* = 1.27 ms; *TR* = 20 s, matrix size = 32 x 32; field of view (*FOV*) = 12 x 12 mm^2^; slice thickness = 40 mm; flip angle = 90°, Gaussian pulse, bandwidth *BW* = 50 kHz). ^1^H *T*_2_* images were acquired with a multi-gradient echo (MGE) sequence (*N*_*R*_ = 32, *TR* = 3 s, matrix size = 32 x 32, *FOV* = 12 x 12 mm^2^, slice thickness = 40 mm, with 12 echo times ranging from 1.98 to 14 ms).

To test the approach in a biological system, we also performed an experiment assessing the signal of the blood substitute Perftoran (OJSC SPF “Perftoran”, Russia, C_22_F_41_N), in an excised rat lung and heart. This *ex vivo* setup was previously used to develop ^129^Xe imaging techniques [26], but was here used to acquire the ^19^F spectrum of Perftoran in the lung vasculature and heart (estimated at a volume of 1.6 ml). Prior to the experiment, a 1 ml Perftoran sample served as the validation standard to determine the gauge factor. The acquisition was made using a simple pulse-FID sequence with 512 and 128 signal averages, and total experiment durations of 43 min and 2 min for the standard and the lung samples respectively.

### Data processing

SNRs were determined in the images by dividing the average amplitude evaluated from the region of interest (ROI) containing fluorine signal by the average rms amplitude determined from a region containing noise only, using Paravision 5.1 scanner software (Bruker BioSpin GmbH, Ettlingen, Germany). Spectra were processed using Matlab (The Mathworks Inc., Natick, MA). The SNR was optimized according to the matched filter condition by applying a line broadening factor equal to the linewidth at full width half maximum (FWHM) [27]. $T_{2}^{*}$ was evaluated according to $T_{2}^{*}={\left(\pi \Delta \nu \right)}^{-1}$, where Δ*ν* is the linewidth in Hz at FWHM. Images were zero-filled to a 64 x 64 matrix and a sine squared window function was applied. In the porous medium experiment with compound C3, a $T_{2}^{*}$ measurement was additionally determined at the ROI of MGE images using scanner software.

## Results

### Effect of line broadening on ^19^F spectra

**Fig 2:**
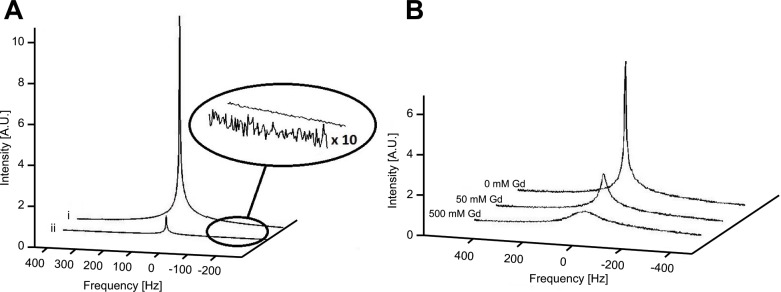
^19^F spectra with varying fluorine concentration and paramagnetic broadening. (A) Spectra of 1000 mM (i) and 100 mM (ii) concentrations of C3. The magnified section shows the scaled noise section. (B) Spectra of C3 (900 mM) with varying concentrations of gadolinium added. The origin of the spectra (0 Hz) in both (A) and (B) corresponds to a laboratory frame of reference on-resonance with the main signal.

Spectral linewidths were found to be similar for different fluorine concentrations without the addition of a line broadening species, as shown in Fig 2A. The effect of paramagnetic broadening through addition of gadolinium on the line shape of C3 (TFA) in different concentrations is shown in Fig 2B. At the lowest TFA concentration (100 mM), the addition of Gd increased the linewidth seven-fold from 7 Hz to 50 Hz (Table 1). Susceptibility broadening, when TFA was added to a porous glass medium, increased spectral lines to a linewidth of 419 Hz (Table 2).

**Table 1:** Calibration factor under paramagnetic broadening.

Concentration	Δ*ν* [Hz]	*T*_2_* [ms]	MRS *Ψ*	MRI *Ψ*	Λ_*S*_ × 10^4^m^1.5^	Λ_*I*_ × 10^4^m^1.5^
100	49.6	6.4	266 ± 24	37 ± 4	7.9 ± 1.7	5.9 ± 0.5
600	29.5	10.8	1888 ± 170	240 ± 65	7.1 ± 1.4	5.0 ± 0.4
1000	29.3	10.9	2667 ± 240	391 ± 109	6.0 ± 1.1	4.9 ± 0.5

Calibration factors derived from experimental SNR data from MR spectroscopy, Λ_*S*_, and imaging, Λ_*I*_, for compound C3 (1000 mM, 600 mM and 100 mM) with paramagnetic broadening through a 50 mM gadolinium. The linewidth is the spectral FWHM.

**Table 2:** Calibration factor under susceptibility broadening.

Concentration	Δ*ν* [Hz]	*T*_2_* [ms]	MRS *Ψ*	MRI *Ψ*	Λ_*S*_ × 10^4^m^1.5^	Λ_*I*_ × 10^4^m^1.5^
212	396	0.80	162 ± 16	13 ± 3	6.2 ± 1.6	7.5 ± 2.1
424	419	0.76	342 ± 31	28 ± 6	6.8 ± 1.7	7.5 ± 1.9

Calibration factors derived from experimental SNR data acquired with MR spectroscopy, Λ_*S*_, and imaging, Λ_*I*_, for C3 (424 mM and 212 mM) with susceptibility broadening through a glass bead medium.

### Determining the ^19^F linewidth

**Fig 3:**
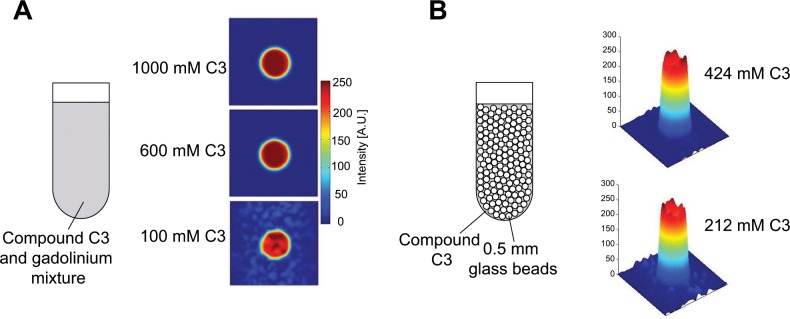
Paramagnetic and susceptibility broadening effect on ^19^F MRI. (A) ^19^F images of C3 (1000 mM, 600 mM, 100 mM) with 50 mM gadolinium added to induce paramagnetic line broadening. Images were acquired with a gradient echo FLASH sequence, *TE* = 1.3 ms, *TR* adapted to allow full relaxation, matrix size = 64 × 64, *FOV* = 1.2 × 1.2 cm^2^, *N*_*R*_ = 1, and slice thickness = 4 cm. (B) ^19^F image intensity plots for C3 (424 mM and 212 mM) in a glass bead medium (sketch) inducing susceptibility line broadening.

As it might be difficult to determine the spatially resolved ^19^F linewidth *in vivo* directly due to the generally low SNR in fluorine MRI, we evaluated whether ^19^F linewidths can be obtained from ^1^H T_2_* intensity fitted MGE images [28]. For the glass bead sample (solution C3 at 424 mM concentration with glass beads in Fig 3B) a fairly uniform value of $T_{2}^{*}$ = 0.72 ± 0.21 ms for ^1^H was found. Generally, $T_{2}^{*}\propto \gamma^{-1}$ if one assumes a static distribution of the molecules within the susceptibility-induced magnetic field gradients as the dominating cause for line broadening. From that we determined a ^19^F relaxation time of $T_{2}^{*}$ = 0.77 ms corresponding to a fluorine linewidth of Δ*ν* = 417 Hz. By comparison, the direct measurement of the fluorine linewidth yielded Δ*ν* = 419 Hz or $T_{2}^{*}$ = 0.77 ms (Table 2).

### Calibration factor

We sought to determine if the calibration factor was approximately constant for a range of solutions and in the presence of field inhomogeneities. *Λ*_*S*_ was calculated from Eq 7, with SNRs estimated from ^19^F MR spectroscopy (Table 3). Across all solutions, the calibration factor was found to remain within a range of 3.9 x 10^4^ m^1.5^ to 5.5 x 10^4^ m^1.5^ despite different chemical structures and an increase in linewidth by up to two orders of magnitude in the presence of field inhomogeneities or due to faster relaxation induced by a paramagnetic agent (Fig 2).

**Table 3:** SNR and calibration factors.

Sample	*n*_*e*_	Concentration [mM]	Gd [mM]	Δ*ν* [Hz]	*Ψ*	*Λ* × 10^4^m^1.5^
**C1**	6	58	0	3.6	700 ± 63	4.6 ± 0.6
**C2**	9	322	0	3.0	5524 ± 497	4.1 ± 0.5
**C3**	3	100	0	8.2	363 ± 31	4.3 ± 0.6
	3	200	0	7.1	723 ± 63	4.0 ± 0.5
	3	400	0	7.2	1499 ± 139	4.2 ± 0.4
	3	600	0	7.0	2152 ± 196	4.0 ± 0.4
	3	800	0	7.3	2983 ± 274	4.2 ± 0.4
	3	1000	0	7.5	3698 ± 348	4.2 ± 0.3
**C3**	3	900	0	7	2418 ± 270	3.9 ± 0.5
	3	900	50	36	1450 ± 143	4.3 ± 0.5
	3	900	500	339	666 ± 67	5.5 ± 0.7

Standard deviations in calibration factor calculations were evaluated from six independent measurements after sample repositioning for each measurement.

### Lowest detectable fluorine concentration

**Fig 4:**
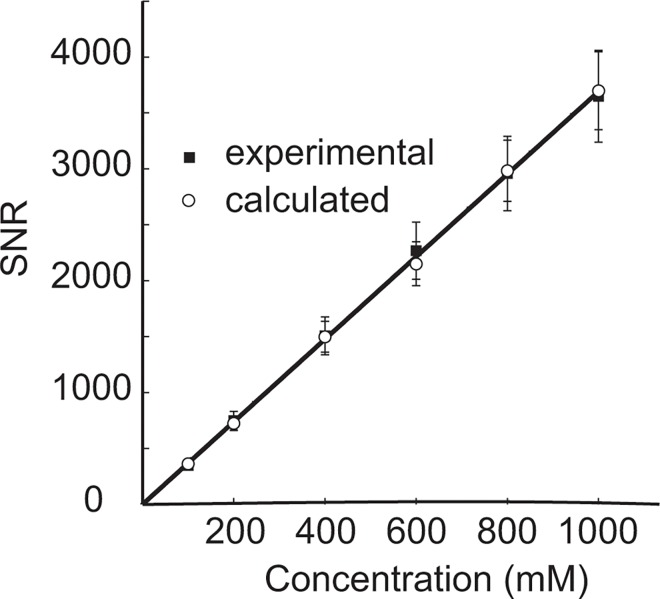
Minimum detectable concentrations in fluorine MRS. Experimental (solid squares) and calculated SNRs (open circles) vs. concentration for compound C3. The calculated SNR was obtained from an average calibration factor using Eq 7. Lines of best fit were extrapolated to determine a minimum detectable concentration assuming a minimum required SNR of 3.5. The fitting of the experimental and derived data resulted in a minimum detectable limit of 0.947 mM and 0.948 mM respectively.

The minimum fluorine concentration detectable for C3 was determined by linear extrapolation of the relationship between SNR and fluorine concentration, based on a method by previous fluorine quantification studies [3,29], using a minimum detectable SNR of *Ψ*_min_ = 3.5 (Fig 4). The SNR detection threshold was chosen according to previous studies, which have used SNR values of 3 [30,31] and 3.5 [3]. The SNR was determined from experiment over a range of concentrations of TFA as well as from calculations using Eq 7 with calibration factor *Λ* = (4.2 ± 1.1) × 10^4^ m^1.5^, the average of the calibration factors reported in Table 3. As expected, both calculated and experimental SNRs showed linear dependence on TFA concentration (r^2^ = 0.99). From extrapolation, the detectable threshold concentration was found to be 0.947 mM for experimental and 0.948 mM for calculated data. This was experimentally verified by measuring the SNR from a 0.4 mM and 1 mM C3 sample (S2 and S3 Figs); 1mM produced an SNR around the minimum detectable limit (*Ψ*_min_ = 3.5), and the 0.4 M sample was found to be below this limit.

### Comparing spectroscopy and imaging

To determine whether the calibration factor would differ if determined from MRI rather than spectroscopic experiments, we performed the experiments in the presence of imaging gradients. The experimental imaging SNR values from MRI and MRS experiments were compared for the samples containing compound C3. The calibration factors in paramagnetically broadened samples were *Λ* = (5.3 ± 0.8) x 10^4^ m^1.5^ when measured by MRI and *Λ* = (7.0 ± 2.5) x 10^4^ m^1.5^ when measured by MRS (average over all TFA concentrations measured, Table 1). With susceptibility broadening, the MRI derived calibration factor was *Λ* = (7.5 ± 2.8) x 10^4^ m^1.5^, as opposed to *Λ* = (6.5 ± 2.3) x 10^4^ m^1.5^ when measured by MRS (average over both TFA concentrations measured).

Finally, we aimed to test if the theoretical estimation also holds in a biological context (S4 Fig). We therefore measured the spectrum from a blood replacement, the perfluorocarbon based Perftoran, within the vasculature of an excised rat lung. We compared the SNR from the lung with the predicted SNR based on an *in vitro* Perftoran sample. The difference between predicted and measured SNR in this case was 2%, although greater differences will be expected *in vivo*, or when comparing different molecules.

## Discussion

In this work, we sought to evaluate the minimum detectable fluorine concentration for a target SNR under a range of conditions that affect the fluorine signal without performing an individual pilot study. A calibration factor was defined that characterizes the specific hardware configuration, including the radiofrequency rf coil and the scanner itself.

It was shown that the calibration factor remained constant for a range of samples with different chemical structures and fluorine concentrations without substantial line broadening, as shown for the gadolinium free data in Table 3.

Even in the presence of inhomogeneous broadening the calibration factor was largely independent of the measurement technique (spectroscopy or imaging), with calibration factor values for both MRS and MRI found to be within error for the majority of data presented in Tables 1 and 2. Therefore the approach proposed here allows for the use of spectroscopic measurements with model compounds to predict the feasibility of planned imaging and spatially localised spectroscopic ^19^F experiments. Also, consistent with previous studies [32], the calibration factor only weakly depended on the window function (exponential decay in spectroscopy, sine squared window function for imaging). The exact choice of the line broadening parameter had a minor effect on the calibration factor, as illustrated in S1 Fig, since the SNR is inversely proportional to the square root of the linewidth and therefore quite robust over a range of broadening values. Furthermore, the calibration factor varied by less than a factor of two when comparing strongly broadened and high resolution data, which is manageable considering the two orders of magnitude difference in linewidth of the sample environments. This factor of two provides an estimate for the accuracy of the predictions achievable with this technique for the different conditions typically encountered *in vivo*.

While the proposed approach is simple to implement, some experimental precautions need to be considered to ensure an accurate estimate of the minimum detectable fluorine concentration. The dwell time, Δ*t*, between data points should be much smaller than $T_{2}^{*}$, otherwise additional systematic errors are introduced due to numerical inaccuracies and possibly filtering out of signal contributions. As long as this condition is fulfilled, the influence of the receiver bandwidth is small. The choice of acquisition coil, e.g. solenoidal or surface coils, will impact on the calibration factor. It may be difficult to estimate the filling factor and coil volume accurately. For coils with a homogeneous rf field, *B*_1_, across the sample volume, this is not an issue since the respective term is factored into *Λ* and therefore experimentally characterized when *Λ* is determined. For surface coils and in particular for sensitivity encoding (SENSE) [33], some form of *B*_1_ correction is necessary [34]. As a simple approximation, the principle of reciprocity [13] could be employed by determining *Λ* at the location with maximum rf field strength, *B*_1_^max^, and scale the threshold concentration by a factor *B*_1_^max^/*B*_1_(**r**), where **r** is the location of interest. For SENSE acquisitions, the hardware dependant signal enhancement is included in *Λ* while pulse sequence or sampling dependant sensitivity factors need to be considered separately.

In general, pulse sequences contribute another factor to the SNR that is largely hardware independent. This factor could be estimated using an analytic expression, as has been done for our FLASH sequence in comparison to the standard single pulse experiment. Alternatively, it is possible to determine such a factor numerically or experimentally. For the latter, a reference scan on a different scanner or spectrometer would be sufficient. Ideally, the reference substance should show comparable relaxation times and linewidths as the compound of interest, and for minimal errors, the reference scan should be performed with similar acquisition parameters as the minimum number of detectable spins calibration experiment.

In our theoretical method we have excluded any temperature dependencies, since our samples were measured in identical conditions in a temperature controlled environment. However, the SNR is proportional to *T*^−1.5^ (Eq 1), where a factor of *T*^−1^ is contributed by the sample magnetization and a factor *T*^−1/2^ is caused by thermal noise. A fluorinated compound administered *in vivo* could result in a SNR decrease of up to 5% compared to a room temperature measurement if coil and sample are independently controlled. By heating the reference sample used for the calibration experiment to the temperature of the environment of the desired fluorine experiment, which is within a narrow range for in vivo studies, such a systematic error could be avoided. If the coil is kept at a constant operating temperature, which could be considerably different from the sample temperature, the temperature difference is absorbed by the calibration factor, hence no systematic error would be expected either.

For *in vivo* applications, *Q* factor changes may be considerable due to the substantial coil loading, but this may be circumvented by using calibration standards that have *Q* factors similar to those typically used in MRI scans. For example, a *Q* factor of 89 was obtained using a CuSO_4_ phantom in a solenoid coil while a *Q* factor of 84 was measured when this coil was loaded in a pre-clinical study at 3 T [35]. Due to the square root dependence of the *Q* value in Eq 2, typical differences observed for the *Q* factor of the loaded resonator, *Q*_*L*_, will only have a minor impact on predictions of the minimum concentration. In principle, if *Q*_*L*_ and the quality factor of an unloaded or empty coil, *Q*_*E*_, are known, they can be used to modify Eq 1 [36]:

$$\Psi =\frac{cN_{A}n_{e}\gamma \hbar^{2}V_{s}}{16}\sqrt{\frac{{Q_{E}K}^{2}{µ}_{0}\omega_{0}^{3}\pi T_{2}^{*}}{V_{c}Fk_{B}^{3}T^{3}}}\sqrt{\frac{Q_{L}}{Q_{E}}}\sin\left(\beta \right)$$ [9]

As stated above, *in vivo* thermal noise should also be accounted for in our approach, since the SNR would be overestimated even when similar *Q* factors are exhibited for a subject compared to a test sample. Ideally, the noise is determined from an *in vivo* SNR experiment, from which the thermal noise contribution could be subsumed into the equations as a constant additional factor.

Quantification of fluorine content *in vitro* is important to extrapolate to labelled cell numbers *in vivo*, and our proposed method could be used to complement this approach. Several studies already calculate a minimum detectable fluorine signal, for example, to convert the fluorine signal of known concentrations to an equivalent number of PFC labelled cells [37]. In certain situations interaction between the fluorine label and host cell can produce shortened T_2_ values, leading to under-estimation of the number of labelled cells [38]. With our proposed model, expected and actual fluorine content could be compared to infer underlying biological processes causing a signal reduction, e.g. cell division.

Although demonstrated specifically for ^19^F experiments, the presented approach is not limited to this nucleus. Providing independent calibrations are performed for different nuclei on a given set-up, our method may be used to predict expected SNRs and minimum detectable concentrations for any nuclei. However, in cases where the line shape of an X-nucleus is not dominated by susceptibility-induced magnetic field gradients, estimation using a ^1^H measurement may not be appropriate and the linewidth or *T*_2_* value should be experimentally verified.

## Conclusion

We presented a simple approach that allows for the prediction of minimum detectable fluorine concentrations in ^19^F MRI experiments. It was demonstrated for a range of experimental conditions that a calibration factor, specific for a given MRS or MRI hardware configuration, can be determined once and then be used to estimate the feasibility of a planned ^19^F MR study. The method provides a framework for the sensitivity estimation of ^19^F experiments and may be most useful during the design of novel fluorine based contrast agents.

## Supporting Information

S1 FigEffect of line broadening (LB) on the SNR.(A) Line broadening with an exponentially decaying window function corresponding to a linewidth of 25 Hz and 50 Hz applied to a sample spectrum of gadolinium doped sample C3, compared with the original spectrum. (B) The optimal line broadening factor was 50 Hz, corresponding to 100% relative LB to FWHM. The SNR and FWHM values follow the expected relation SNR $\propto {T_{2}^{*}}^{0.5}\propto {FWHM}^{-0.5}$ (Eq 1).(EPS)

S2 Fig Representative ^1^H MGE image used to calculate *T*_2_*. A proton image from a MGE scan is shown, (*TE* = 1.98 ms), which was part of a series of images with different echo times used to obtain a *T*_2_* value for comparison with spectral *T*_2_* (12 echo images, 1 ms spacing).
(EPS)

S3 Fig Detection limits at low fluorine SNR regimes. Spectra showing the signal from a 1 mM and 0.4 mM TFA (C3) sample. An SNR of 5.8 was measured for the 1 mM sample, although the linewidth was 5.1 Hz rather than between 7 and 8 Hz used for the line of best fit. Accounting for this in Eq 5 gives an SNR of 3.8, close to the predicted 3.5. For the 0.4 mM sample, 8 averages were needed to obtain a signal, showing a single scan SNR of 1.7, i.e. below the stated detection limit.
(EPS)

S4 Fig A complex fluorine signal from the lung vasculature. To demonstrate the application of the approach, we estimated the SNR from the synthetic blood Perftoran, which carries 41 fluorine atoms and a complex line shape, in the vasculature of an excised rat lung. To estimate the gauge factor, a standard spectrum of Perftoran in a 1 ml test tube was measured. The areas in the spectra from which noise and the signal were estimated, are indicated. The fluorine signal from the lung vasculature is here magnified by a factor of 20.
(EPS)

S1 Text Derivation of Theoretical SNR and description of relaxometry. (DOCX)
