# Supplementary material for: Probe-Specific Procedure to Estimate Sensitivity and Detection Limits for 19F Magnetic Resonance Imaging
Source: PLoS One. 2016 Oct 11;11(10):e0163704. doi: 10.1371/journal.pone.0163704 (PMC5058479; doi:10.1371/journal.pone.0163704)
Supplement: S1 Text — (DOCX) [file pone.0163704.s005.docx]

**S4. Derivation of Theoretical SNR and relaxometry information**

**Theoretical SNR**

In the formulation of the signal-to-noise ratio (SNR) described by Abragam [[1](#_ENREF_1)] and adapted to pulsed magnetic resonance, where the receiver bandwidth is replaced by the bandwidth of a matched filter [[2](#_ENREF_2)], the theoretically achievable SNR,$\Psi$, in a single pulse nuclear magnetic resonance experiment can be approximated by

$\Psi= K\eta_{c}M_{0}\sqrt{\frac{\mu_{0}Q\omega_{LC}V_{c}\pi T_{2}^{*}}{4Fk_{B}T_{c}}}\sin(\beta)$. [1]

Here $k_{B}$ is the Boltzmann constant, $M_{0}$ the nuclear magnetization of the sample at thermal equilibrium, $T_{c}$ the coil temperature, $V_{c}$ the coil volume, $F$ the pre-amplifier noise figure (which is generally constant for the frequency range covered by a given nucleus [[3](#_ENREF_3)]), $\mu_{0}$ the permeability of free space,$Q$ the quality factor of the detection circuit, $\omega_{LC}$ the frequency of the resonant circuit, $T_{2}^{*}$ the effective transverse relaxation time, and $\beta$ the flip angle associated with the excitation pulse. $K$ is a numerical factor introduced by Hoult and Richards to account for the specific receiving coil geometry [[4](#_ENREF_4)]. For the following in vitro experiments, Q should be similar and small differences in loading will not be significant due to the square root dependence. However, for in vivo situations the coil's loaded quality factor, $Q_{L}$, will be significantly different from the empty quality factor, $Q_{E}$ thus affecting the value of $\Psi$ in Eq. 1. Therefore, the incorporation of the $Q_{L}$ factor into the model for the in vivo translation is significant and will be described in the Discussion section. The filling factor $\eta_{c}$ for an ideal solenoid volume coil can be approximated as

$\eta_{c}\approx\frac{V_{s}}{2V_{c}}$ [2]

where $V_{s}$ is the sample volume. For spin 1/2 nuclei, such as ^19^F, in the high temperature limit $M_{0}$ is given by

$M_{0}=\frac{N\gamma^{2}\hbar^{2}B_{0}}{4k_{B}T_{s}}$ [3]

where $N$ is the number of contributing spins per unit volume, $\hbar$ the reduced Planck constant,$B_{0}$ is the magnetic field strength, 𝛾 the gyromagnetic ratio, and $T_{s}$ the sample temperature. The term $\sin(\beta)$ in Eq. 1 represents the contribution to SNR associated with the pulse sequence - in this case, a single-pulse experiment with detection of the free induction decay (FID). Eq. 1 is valid when a matched filter is applied, which can easily be implemented by multiplying the time-domain signal by an exponential window function with time constant $T_{2}^{*}$ prior to Fourier transformation [[2](#_ENREF_2)]. We can further assume that $\omega_{LC}\approx|\omega_{0}|$with $\left| \omega_{0} \right|=|\gamma_{0}B_{0}|$, as the resonant frequency of the circuit is tuned to the Larmor frequency of the ^19^F signal. As the coil temperature, *T_c_*, should not differ significantly from the sample temperature, *T_s_*, we assume that *T_c_ ≈* *T_s_ = T*. The number of contributing spins in a sample per unit volume, $N$, is defined as $N= N_{s}V_{s}^{-1}$, where the total number of contributing spins in a sample is given by

$N_{s}=cN_{A}n_{e}V_{s}$. [4]

where $c$ is the concentration in units of mol/m^3^, $V_{s}$ the sample volume, $n_{e}$ the number of equivalent fluorine spins per molecule with the same chemical shift, and $N_{A}$ the Avogadro constant. Thus the SNR can be expressed as:

$\Psi= \frac{cN_{A}n_{e}\gamma\hbar^{2}V_{s}}{16}\sqrt{\frac{K^{2}\mu_{0}Q\omega_{0}^{3}\pi T_{2}^{*}}{V_{c}Fk_{B}^{3}T^{3}}}\sin(\beta)$ . [5]

An explicit equation for the fluorine marker concentration, $c$, required to obtain a target SNR is obtained after rearranging Eq. 5:

$c = \frac{16\Psi}{N_{A}n_{e}\gamma\hbar^{2}V_{s}}\sqrt{\frac{V_{c}Fk_{B}^{3}T^{3}}{{K^{2}\mu}_{0}Q\omega_{0}^{3}\pi T_{2}^{*}}}\frac{1}{\sin(\beta)}$ . [6]

$\boldsymbol{T}_{\boldsymbol{1}}$**relaxation values**

^19^F spin-lattice relaxation times, $T_{1}$, were measured using a spectroscopic inversion recovery sequence with eight logarithmically spaced time delays between 0.1 s and 20 s. It was verified that all samples used in this work have a $T_{1}$ less than 4 s, which is at least five times shorter than the experiment repetition time. Hence $T_{1}$ is not considered as an affecting factor in the sensitivity discussion.

$\boldsymbol{T}_{\boldsymbol{2}}^{\boldsymbol{*}}$ **relaxation values**

Using a MGE proton scan, $T_{2}^{*}$ measurements were obtained from the following representative mean intensity and standard deviations values from the proton ROI (from Paravision 5.1 Bruker software):

| **Echo time (ms)** | **Mean intensity value (arbitrary units)** | **Standard deviation (arbitrary units)** |
| --- | --- | --- |
| 1.98 | 3365321 | 378061 |
| 3.07 | 486768 | 232061 |
| 4.16 | 188562 | 118504 |
| 5.25 | 152700 | 83045 |
| 6.34 | 102825 | 77880 |
| 7.43 | 110359 | 84296 |
| 8.52 | 125295 | 65214 |
| 9.61 | 88936 | 58332 |
| 10.7 | 60331 | 30826 |
| 11.8 | 33100 | 13394 |
| 12.9 | 31863 | 17284 |
| 14 | 37960 | 17183 |

1. Abragam A. The Principles of Nuclear Magnetism. 1^st^ ed. Clarendon Press; 1961

2. Ernst RR, Bodenhausen G and Wokaun A. Principles of Nuclear Magnetic Resonance in One and Two Dimensions. 1^st^ ed. Oxford University Press; 1987.

3. Nordmeyer-Massner JA, De Zanche N, and Pruessmann KP. Noise figure characterization of preamplifiers at NMR frequencies. J Magn Reson 2011; 210: 7-15.

4. Hoult DI and Richards RE. The signal-to-noise ratio of the nuclear magnetic resonance experiment. J Magn Reson 1976; 24:71-85.
